# Nanoparticles With Affinity for α-Synuclein Sequester α-Synuclein to Form Toxic Aggregates in Neurons With Endolysosomal Impairment

**DOI:** 10.3389/fnmol.2021.738535

**Published:** 2021-10-20

**Authors:** Peizhou Jiang, Ming Gan, Shu-Hui Yen, Dennis W. Dickson

**Affiliations:** ^1^Department of Neuroscience, Mayo Clinic, Jacksonville, FL, United States; ^2^Department of Laboratory Medicine and Pathology, Mayo Clinic, Jacksonville, FL, United States

**Keywords:** α-synuclein, aggregation, endolysosomal impairment, Parkinson’s disease, GBA

## Abstract

Parkinson’s disease (PD) is one of the most common neurodegenerative diseases. It is characterized pathologically by the aggregation of α-synuclein (αS) in the form of Lewy bodies and Lewy neurites. A major challenge in PD therapy is poor efficiency of drug delivery to the brain due to the blood–brain barrier (BBB). For this reason, nanomaterials, with significant advantages in drug delivery, have gained attention. On the other hand, recent studies have shown that nanoparticles can promote αS aggregation in salt solution. Therefore, we tested if nanoparticles could have the same effect in cell models. We found that nanoparticle can induce cells to form αS inclusions as shown in immunocytochemistry, and detergent-resistant αS aggregates as shown in biochemical analysis; and nanoparticles of smaller size can induce more αS inclusions. Moreover, the induction of αS inclusions is in part dependent on endolysosomal impairment and the affinity of αS to nanoparticles. More importantly, we found that the abnormally high level of endogenous lysosomotropic biomolecules (e.g., sphingosine), due to impairing the integrity of endolysosomes could be a determinant factor for the susceptibility of cells to nanoparticle-induced αS aggregation; and deletion of GBA1 gene to increase the level of intracellular sphingosine can render cultured cells more susceptible to the formation of αS inclusions in response to nanoparticle treatment. Ultrastructural examination of nanoparticle-treated cells revealed that the induced inclusions contained αS-immunopositive membranous structures, which were also observed in inclusions seeded by αS fibrils. These results suggest caution in the use of nanoparticles in PD therapy. Moreover, this study further supports the role of endolysosomal impairment in PD pathogenesis and suggests a possible mechanism underlying the formation of membrane-associated αS pathology.

## Introduction

Parkinson’s disease (PD) is the second most common age-related neurodegenerative disease worldwide (Mhyre et al., 2012). Pathologically, PD is characterized by the loss of dopaminergic neurons in the substantia nigra and by intraneuronal α-synuclein (αS) aggregates in the form of Lewy bodies and Lewy neurites (Mhyre et al., 2012). Although the restoration of dopaminergic neurotransmission and alleviation of burden of αS have been considered as two key strategies for PD therapy (Meissner et al., 2011; Mhyre et al., 2012), there is still no cure for this disease. This is in part due to the presence of the blood–brain barrier (BBB), which reduces the efficiency of drug delivery to the brain. It may also be due to a decreased biological activity of the drug resulting from enzymatic degradation or other factors encountered by the drug during its delivery (Gavhane and Yadav, 2012; Hersh et al., 2016; Raza et al., 2019). In this regard, nanoparticles, due to their unique properties in size and biodegradability (Mahapatro and Singh, 2011; Shang et al., 2014; Hoshyar et al., 2016), as well as their BBB permeability and drug loading capacity (Shen et al., 2017; Teleanu et al., 2018), have attracted attention as a drug-delivery approach in PD (Leyva-Gomez et al., 2015; Lafuente et al., 2019).

On the other hand, some nanoparticles that have been suggested for drug delivery (Murthy, 2007) have been shown to induce fibrillization of aggregation-prone proteins (Linse et al., 2007; D’Onofrio et al., 2020). For example, recombinant soluble αS in salt solution has been induced to form aggregates upon the addition of nanoparticles (Alvarez et al., 2013; Mohammadi and Nikkhah, 2017; Tahaei Gilan et al., 2019). Some of those aggregates were cytotoxic to cultured neuronal cells (Tahaei Gilan et al., 2019). Since endocytosis is a major pathway in nano-based drug delivery to cells (Behzadi et al., 2017), we were interested in determining if nanoparticles can directly interact with cytoplasmic αS in neuron cells, and if this interaction can induce αS to form neurotoxic aggregates. Our results not only pointed out a potential risk of nanoparticles in PD treatment but also revealed a possible mechanism underlying the formation of membrane-associated αS pathology in PD.

## Materials and Methods

### Cell Culture and Maintenance

Cell cultures in this study included transfectants derived from human H4 neuroglioma: “H4/V1S:SV2,” “H4/CBD-V1S:SV2-CBD,” “H4/V1S:SV2/LAMP1-eCFP/mCherry-galectin-3,” and a transfectant from BE(2)-M17 neuroblastoma cells—“3D5.” The purpose of using each cell line was described in the section of Results. All cell lines were maintained in OPTI-MEM (Invitrogen) medium containing 10% fetal bovine serum (Invitrogen) at 37°C with 5% CO_2_ and 100% humidity. For live cell imaging with confocal microscopy, cells were cultured in Nunc^®^ Lab-Tek^®^ II chambered coverglass (Sigma-Aldrich). For the differentiation of human dopaminergic cell line BE(2)-M17-derived cells, the medium was replaced with Neurobasal medium (Invitrogen), 2% B-27 supplement (Invitrogen), 2 mM L-glutamine (Sigma-Aldrich), and 10 μM retinoic acid (Sigma-Aldrich).

### Lentiviral Plasmids and Virus Preparation

Lentiviral plasmids carrying LAMP1-eCFP and mCherry-galectin-3 were described previously (Jiang et al., 2017). The lentiviral vector for CRISPR-Cas9 knockout of GBA1 was designed by VectorBuilder Inc. (Supplementary Figure 2). The sequences of two single guide RNAs (sgRNAs) were “AGACCAATGGAGCGGTGAAT” and “TGTGGTGAGTACT GTTGGCG,” respectively. The protocols used for the preparation of lentivirus carrying genes of interest were the same as described previously (Jiang et al., 2013).

### Nanoparticle Preparation

Commercially available nanoparticles were Gold (Nanocs), TiO_2_ (US Research Nanomaterials), ZnO (Inframat), Fe_3_O_4_ (US Research Nanomaterials), and SiO_2_ (US Research Nanomaterials). The Alexa Fluor^TM^ 647-labeled SiO_2_ nanoparticles were customized product (30 nm) from Nanocs. The chitin nanoparticles were customized (80 nm) from Nanoshel. To prepare fresh concentrated nanoparticle solutions, the original powder was weighed and added into 1 × PBS, then sonicated for 5 min at maximal power (Sonicator 3000, Misonix). The concentrated nanoparticle solution was further diluted into culture media to make final nanoparticle-supplemented media, which were sonicated for at least 1 min before cell treatment.

### Nanoparticle Treatment and Quantification of Induced αS Inclusions in Cells

Cells were plated on coverglass with 8-well culture chamber at the same density overnight, then treated with different nanoparticles. The next day, the cells were exposed to 1 mM lysosomotropic detergent [L-leucyl-L-leucine methyl ester (LLME)] (Cayman Chemical) for 1–2 h to induce endolysosomal rupture. In parallel, sibling cultures without nanoparticles or LLME treatment were set as two different negative controls. After fixation with 4% paraformaldehyde (PFA), cells with and without αS inclusions were evaluated and photographed under a confocal microscope (Zeiss LSM 510, Carl Zeiss MicroImaging). For measuring the ratio of cells bearing αS inclusions, five fields (upper right, upper left, center, lower right, and lower left) with at least 90 cells were selected from each group for cell count.

### Separation of Detergent-Soluble and Insoluble Fractions

Neuroblastoma BE(2)-M17D-derived cell model—3D5 (Ko et al., 2008) were differentiated and induced to express human wild-type αS, then exposed to media supplemented with and without SiO_2_ nanoparticles for 1 day, followed by the induction of endolysosomal membrane rupture for 1–2 h. Cells were then harvested for protein extraction by extraction buffer [1% Triton X-100 (Tx) and 1% (v/v) protease inhibitor cocktail (Sigma) in 1 × PBS] to obtain Tx-soluble and Tx-insoluble fractions following a previous protocol (Bae et al., 2015). The same amount of proteins from different groups was mixed with loading buffer and then resolved by sodium dodecyl–sulfate polyacrylamide gel electrophoresis (SDS–PAGE) followed by the Western blotting.

### Assay Comparing αS Binding Affinity for Different Nanoparticles

The method for detecting the binding affinity of nanoparticles to αS was similar to that reported in a previous study (Hata et al., 2014) with minor modifications. Freshly prepared recombinant αS solution was mixed with different nanoparticles to get a final concentration of 0.5 μg/μl for αS and 200 μg/ml for both SiO_2_ and chitin, respectively. A tube of αS solution with the same concentration of αS but without mixing with any nanoparticle was included as a control (Con). All samples were incubated at 37°C for 1 h with constant rotating. The αS protein bound to nanoparticles was isolated by centrifugation at 30,000 × *g* for 20 min at 4°C. The top layer of supernatant was removed to a new tube for measuring the concentration remained in solutions. The pellets containing a mixture of nanoparticles and particle-bound αS were washed with 1 ml PBS and centrifuged again for three times to remove residual unbound αS protein, then mixed with 10% SDS buffer, followed by the addition of an equal amount of Laemmli sample buffer (Bio-Rad Laboratories) and boiled for 5 min at 95°C. The boiled samples were centrifuged, and the supernatants were used for SDS–PAGE. The gels were subjected to silver staining to show the bound αS in each group. A tube containing αS solution was saved before mixing with nanoparticles and used as a negative control.

### Sphingosine Measurement in Cell Cultures

Pellets of cultured cells (comparable cell number per group) were resuspended in 1 × PBS and then lysed by ultrasonication four times, followed by centrifugation at 1,500 × *g* for 10 min at 4°C. Supernatants collected from cultured cells were used for measuring the concentration of sphingosine according to the manufacturer’s instructions (Sphingosine ELISA Kit Lifespan Biosciences). Briefly, samples were added to a plate, followed by the addition of detection reagent A and 1 h of incubation at 37°C. After incubation, the reagents in the plate were removed and the plate was washed completely with buffer and then loaded with detection reagent B for 45 min of incubation at 37°C. At the end of incubation, sample wells were emptied and washed again, then loaded with TMB substrate for 10–20 min of incubation at 37°C, followed by the addition of stop solution and the measurement of optical density by a microplate reader (SpectraMax Paradigm, Molecular Devices).

### Immunocytochemistry

Cells were rinsed with 1 × PBS, fixed in 4% PFA, and permeabilized with 0.1 M Tris-buffered saline (TBS; pH 7.6) containing 0.5% Triton X-100 for 5 min, then blocked with 3% goat serum in TBS, incubated with the primary antibody in TBS containing 1% goat serum overnight at 4°C and then with the secondary antibody for 1 h at room temperature. Immunolabeled cells were mounted in VECTASHIELD^®^ antifade mounting media with or without DAPI (Vector Laboratories), then examined under a confocal microscope. Primary antibodies included mouse against GBA (Abcam) and mouse against HA (Sigma), and the secondary antibodies include the Alexa Fluor 568 and the 647 anti-mouse (Thermo Fisher Scientific).

### Induction of Intracellular αS Inclusions by Exogenous αS Fibrils

H4/V1S:SV2/LAMP1-eCFP/mCherry-galectin-3 cells with GBA1 deletion (GBA1-) or without (WT) were treated with mature fibrils derived from recombinant αS fused with HA tag (αSHA) as described previously (Jiang et al., 2008). Such αSHA fibrils were preincubated at 4°C for 2 days to facilitate the endocytosis seeding pathway because such incubation can significantly reduce the capability of αS fibrils in direct penetration of cell membrane according to our previous study (Jiang et al., 2017). Cells were fixed when GBA- cells formed enough seeded αS inclusions under confocal microscope. A portion of sibling cells from each group was subjected to immunocytochemical staining with antibody against HA tag to demonstrate the distribution of exogenous αSHA-fibrils (shown as Alexa Fluor^TM^ 647). Another portion of cells was subjected to electron microscopy (EM) samples process and subsequent conventional EM and immuno-EM. Immuno-EM was performed by immunolabeling Venus (orb334993, Biorbyt) and HA tag with a gold of 15 nm (25806, EMS) and 2 nm (25125, EMS) in samples, respectively.

### Electron Microscopy and Immunoelectron Electron Microscopy

Cells for transmission EM were fixed with 2% glutaraldehyde, 2% PFA in 0.1 M PBS; cells for Immunoelectron Electron Microscopy (immunoEM) were fixed with 4% PFA in 0.1 M PBS. For EM, cells were postfixed in 1% OsO_4_; washed three times in distilled water; stained with 1% uranyl acetate in 50% ethanol; and dehydrated with 70, 80, 95, and 100% ethanol sequentially. The cells were then treated with propylene oxide, infiltrated, and embedded in Epon 812 (Polysciences). For immunoEM, the cells were dehydrated in 30, 50, 70, and 90% ethanol, sequentially, then 90% ethanol-LR White (1:1) and 90% ethanol-LR White resin (1:2). They were then infiltrated and embedded in pure LR White. Ultrathin sections were cut from the Epon 812 or LR White-embedded samples by Leica Ultramicrotome. Ultrathin sections were examined after counterstaining with uranyl acetate and lead citrate. The sections were examined and photographed with a Philips 208S electron microscope.

### Statistical Analysis

Data from at least three sets of independent experiments were analyzed by one-way ANOVA with Dunnett’s *post hoc* test or Student’s *t*-test for the comparison of groups >3 and (=2, respectively, to determine statistical significance.

## Results

### Internalization of Different Nanoparticles With Similar Size Induces the Formation of αS Inclusions in a Cell Model With Endolysosomal Impairment

First, we want to know if nanoparticles from different materials are able to induce the formation of αS aggregates within cells. For this purpose, we tested nanoparticles that have been shown to promote αS to aggregate in salt solutions through direct interaction or to accumulate in cells through an indirect mechanism (Alvarez et al., 2013; Joshi et al., 2015; Xie and Wu, 2016; Mohammadi and Nikkhah, 2017; Tahaei Gilan et al., 2019; Khodabandeh et al., 2020). The nanoparticles tested were as follows: SiO_2_, Ti_2_O_3_, Fe_2_O_3_, and ZnO. To maximize the comparability of results from different nanoparticles, only those with similar size (∼30 nm) were used.

The cell model used for the evaluation of nanoparticle-induced αS aggregation was derived from H4 neuroglioma cell line (ATCC^®^ HTB-148^TM^). H4 cells were transfected to stably express the N-terminal half of Venus YFP tagged to αS (V1S) and C-terminal half of Venus YFP tagged to αS (SV2). This transfectant, referred to as H4/V1S:SV2, is useful for monitoring the aggregation of αS in live cells in real time. Because binding between V1S and SV2 will reconstitute YFP fluorescence, the brightness of fluorescence emitted can be used to estimate the extent of αS aggregation (Jiang et al., 2017). H4/V1S:SV2 cells growing in eight-well-chambered culture coverglass were treated with the same concentration of different nanoparticles (30–40 μg/ml) and then observed under confocal microscope daily to monitor the formation of αS inclusions.

Our results showed no evidence of αS inclusions in cells after 1 week of nanoparticle treatment. Since using H4/V1S:SV2 cells to visualize αS aggregation induced by exogenous seeds is a well-established experiment in our laboratory (Jiang et al., 2017), and the time for the induced αS inclusion to appear in this cell line has never been more than 3 days, we deduced that such negative results could be due to insufficient nanoparticles escaping the endocytic pathway to interact with cytoplasmic αS. If this is the case, cells with impaired endolysosomes should allow more nanoparticles to enter the cytoplasm. Since lysosomal dysfunction has been suggested to be an important pathogenic mechanism in PD (Dehay et al., 2010, 2012; Moors et al., 2016), we wondered if αS aggregation could be induced by nanoparticles in cells with endolysosomal impairment. For this purpose, the lysosomotropic detergent LLME that impairs endolysosomal function by irreversible accumulation in acidic compartments, leading to damage of endolysosomal membranes (Uchimoto et al., 1999) was chosen.

**FIGURE 1:**
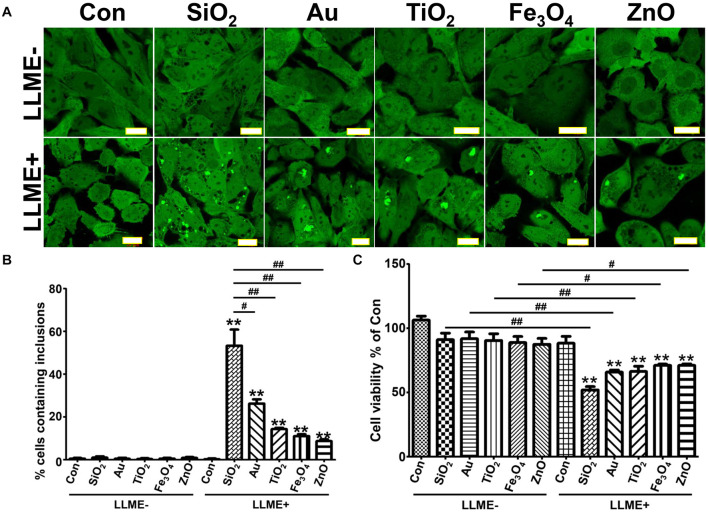
Nanoparticles of different materials induce the formation of αS inclusions in cells with endolysosomal impairment upon internalization. H4/V1S:SV2 cells were treated with the same concentration of different nanoparticles of similar size (∼30 nm). A day later, cells were further treated with 1 mM LLME for 1–2 h to induce endolysosomal membrane rupture, sibling cultures without such treatment were set as negative control (LLME-). **(A)** The representative images from different group of cells were taken under confocal microscope. Scale bar: 20 μm. **(B)** Results of counting inclusion-bearing cells for different groups were statistically analyzed and shown as a bar graph. **(C)** Cells in 12-well plates were washed and harvested for cell counting using a hemocytometer, and the results were statistically analyzed and shown as a bar graph. Error bars represent the standard error of the mean (**p < 0.01, compared with Con in LLME-treated groups (LLME +); ^#^*p* < 0.05, ^##^*p* < 0.01, comparing subsets linked by line, *n* = 3).

Cells treated with different nanoparticles for 1 day were exposed to 1 mM LLME to disrupt the integrity of endolysosomal membranes. As expected, after 1–2 h in LLME, all nanoparticle-treated cells developed αS inclusions. We observed differences in the ability of different nanoparticles to induce the formation of αS inclusions (Figures 1A,B). Moreover, nanoparticle-treated cultures exposed to LLME had fewer cells compared to those without such exposure, and the difference was statistically significant. Exposing cells to LLME in the absence of nanoparticles for 1–2 h did not result in either formation of αS inclusions or cell death, indicating that the nanoparticle-induced αS inclusions are cytotoxic (Figure 1C).

Next, we explored if nanoparticle-induced αS inclusions were associated with the rupture of endolysosomal membranes. For this study, we focused on SiO_2_ nanoparticles, because cells treated with SiO_2_ had the most αS inclusions (Figures 1A,B). Moreover, SiO_2_ nanoparticles are less expensive, and they are the most common nanoparticle used in humans, such as in cosmetics (Napierska et al., 2010; Murugadoss et al., 2017).

### Endolysosomal Impairment Plays an Essential Role in the Formation of Nanoparticle-Induced αS Inclusions

**FIGURE 2:**
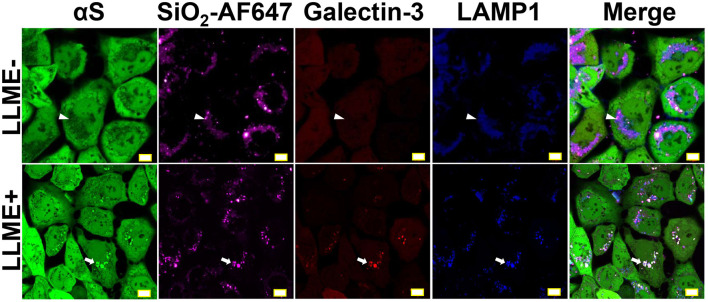
Endolysosomal membrane rupture plays an essential role in the formation of nanoparticle-induced αS inclusions. H4/V1S:SV2/LAMP1-eCFP/mCherry-galectin-3 cells were exposed to media supplemented with the same concentration of Alex Fluor^TM^ 647-labeled SiO_2_ nanoparticles. A day later, cells were treated with 1 mM LLME to induce endolysosomal membrane rupture; sibling cultures without such treatment were set as negative control (LLME-). After 1–2 h, cells were subjected to imaging to show the distribution of intracellular nanoparticles (Alex Fluor^TM^ 647), αS inclusions (accumulated Venus), endolysosome (eCFP), and ruptured endolysosomes (punctuated mCherry-galectin-3), and their colocalization (denoted by white arrows and arrow-heads) under a confocal microscope. Scale bar: 10 μm.

Since the accumulation of galectin-3 on endolysosomal membrane is an indicator of endolysosomal rupture (Flavin et al., 2017), we introduced mCherry-tagged galectin-3 (mCherry-galectin-3) and eCFP-tagged LAMP1 (LAMP1-eCFP) into H4/V1S:SV2 cells to generate a new cell line referred to as H4/V1S:SV2/LAMP1-eCFP/mCherry-galectin-3. Cells from these transfectants were treated with Alex Fluor^TM^ 647-labeled SiO_2_ nanoparticles (SiO_2_-AF647) for 1 day, then exposed to LLME. Under confocal microscopy, we found that all induced αS inclusions were closely associated with galectin-3 and LAMP1 as reflected by the colocalization of Venus, mCherry, and eCFP (arrows in Figure 2). In contrast, cells treated with SiO_2_ nanoparticles alone showed the retention of nanoparticles in endolysosomes, no rupture of endolysosomes, and no αS inclusions, as reflected by colocalization between Alex Fluor^TM^ 647 and eCFP without the accumulation of mCherry and Venus (denoted by arrow heads in Figure 2). These results strongly supported that endolysosomal membrane rupture may play a role in nanoparticle-induced formation of αS inclusions.

### Nanoparticles Induce the Formation of Intracellular αS Inclusions in a Size-Dependent Manner

**FIGURE 3:**
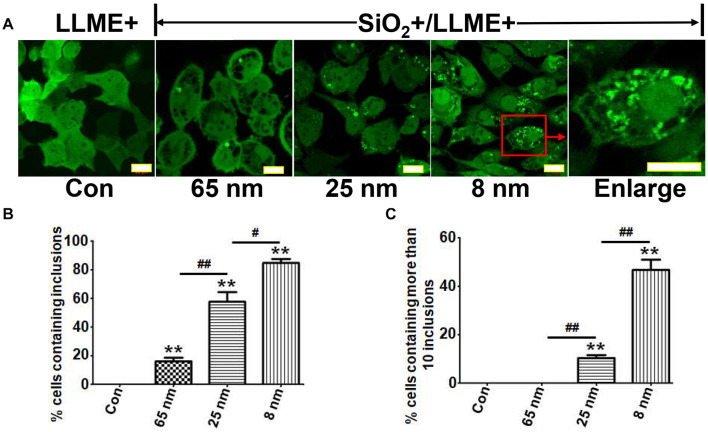
Nanoparticles induce the formation of intracellular αS inclusions in a size-dependent manner. H4/V1S:SV2 cells were treated with the same concentration of SiO_2_ nanoparticles with different sizes (8, 25, and 65 nm), respectively. Sibling cultures without SiO_2_ but with LLME treatment were set as negative control (LLME+). **(A)** The representative images from a different group of cells were taken under a confocal microscope. Scale bar: 10 μm. **(B)** The ratios of cells bearing αS inclusions to total cells for each group were statistically analyzed and shown as a bar graph. **(C)** The ratios of cells containing more than 10 inclusions of αS to total inclusion-bearing cells for each group were statistically analyzed and shown as a bar graph. Error bars represent the standard error of the mean (**p < 0.01, compared with Con; ^#^*p* < 0.05, ^##^p < 0.01, comparing subsets linked by line, *n* = 3).

It is known that the size of nanoparticles can highly influence their *in vivo* pharmacokinetics and cellular interaction (e.g., cellular uptake, biodistribution, and circulation half-life) (Hoshyar et al., 2016). Therefore, we studied the influence of nanoparticle size on the formation of αS inclusions. H4/V1S:SV2 cells were treated with SiO_2_ nanoparticles of three different sizes (8, 25, and 65 nm), respectively, then exposed to LLME. For this experiment, the concentration of nanoparticles was 200 μg/ml, which is over-saturated because nanoparticles of all three different sizes at lower concentration did not show a consistent proportion of αS inclusion-bearing cells. Using saturation levels of nanoparticles excludes the possibility that observed differences between different-sized nanoparticles were due to the presence of more small nanoparticle particles than large ones at a given concentration. Our results showed that the ratio of cells containing αS inclusions to total cells was about 16, 58, and 85%, respectively, for cells treated with nanoparticles of 65, 25, and 8 nm in size (Figures 3A,B). In addition, we found that in cultures treated with smaller nanoparticles, more cells contained multiple αS inclusions. The ratios of cells containing more than 10 inclusions of αS to total inclusion-bearing cells were 0, 13, and 55%, respectively, for those treated with nanoparticles of 65, 25, and 8 nm in size (Figures 3A,C). These results indicated that nanoparticles induce the formation of intracellular αS inclusions in a size-dependent manner.

### Nanoparticles Induce αS to Form Detergent-Insoluble Aggregates Accompanied With an Increase in Pathological Form of αS

**FIGURE 4:**
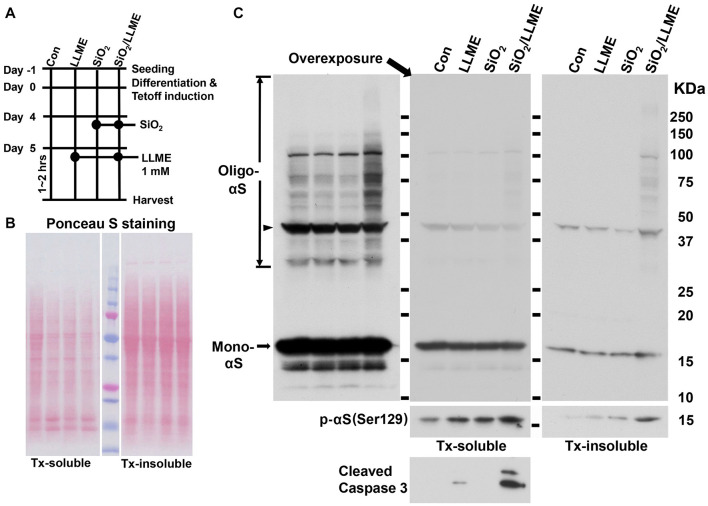
Nanoparticles promote αS to form detergent-insoluble aggregates accompanied with an increase of pathological form of αS. **(A)** shows the experimental design. **(B)** Polyvinylidene difluoride (PVDF) membranes with transferred proteins were stained with Ponceau S to show that the amount of protein in different lanes was comparable. **(C)** After Ponceau S destaining and milk blocking, the blots were subjected to immunoblotting with antibodies against αS (610786, BD Biosciences), phosphorylated αS at serine 129 (pSyn #64, FUJIFILM Wako), and cleaved caspase 3 (9661, Cell Signaling), respectively. Monomeric and oligomeric αS were denoted by arrows; non-specific bands between 37 and 50 KDa were denoted by arrowhead.

Next, we determined if nanoparticle-induced aggregates contain detergent-insoluble αS. Because H4/V1S:SV2 cells express a Venus tag-fused αS, to rule out the possible promotive effect of Venus on the formation of detergent-resistant αS, we used a cell line, 3D5 (Ko et al., 2008), expressing unlabeled αS. Cells of this model were derived from a neuroblastoma BE(2)-M17 cell line. They inducibly express wild-type human αS through a Tetoff mechanism and display neuronal phenotypes upon retinoic acid-induced differentiation (Ko et al., 2008). 3D5 cells were differentiated and induced to express αS in media with retinoic acid, but without Tet (see Figure 4A). On the 5th day, half of the cultures were treated with SiO_2_ nanoparticles for 24 h. Subsequently, cultures with and without nanoparticle treatment were treated with LLME for 1–2 h. The other half served as control. The four groups of cells were referred to as Con (without any treatment), LLME (treated with LLME only), SiO_2_ (treated with SiO_2_ nanoparticles only), and SiO_2_/LLME (treated with both SiO_2_ nanoparticles and LLME), respectively. They were harvested for protein extraction to separate Triton detergent (Tx)-soluble and insoluble fractions. Both fractions were then analyzed by SDS–PAGE and Western blotting for the detection of αS. The results showed that SiO_2_/LLME had the most αS oligomers in Tx-soluble fraction; and only SiO_2_/LLME contained αS aggregates in Tx-insoluble fractions (Figure 4C). We further tested if phosphorylation on those αS aggregates occurs during the treatment. Results showed that the form of αS phosphorylated at serine 129 was also evidently increased in the group of SiO_2_/LLME in both fractions (Figure 4C). Therefore, in the presence of LLME, nanoparticles can induce our cell model to form detergent-insoluble αS aggregates, which is accompanied by an increase of pathological form of αS (phosphorylation at serine 129) (Bernal-Conde et al., 2019). Moreover, immunoblotting (Figure 4C) demonstrated the presence of higher cleaved Caspase 3 in SiO_2_/LLME than other samples, suggesting the formation of αS aggregates was associated with apoptotic cell death.

### The Binding Affinity of Nanoparticles to αS Determines Its Capability to Induce αS Aggregation in Cells

**FIGURE 5:**
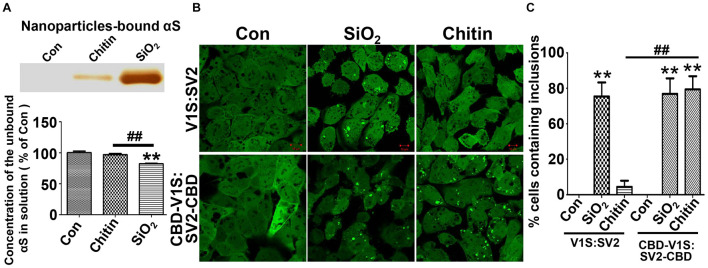
The binding affinity of nanoparticles to αS determines its capability to induce the formation of αS inclusions. **(A)** Results of silver staining demonstrated that more αS was sequestered by SiO_2_ than chitin nanoparticles (see the top panel); such sequestration occurred at the expense of soluble αS. The statistical analysis of unbound αS remained in nanoparticle-deprived solution was shown as a bar graph at the bottom panel. **(B)** The representative images were taken under a confocal microscope from “H4/CBD-V1S:SV2-CBD” and “H4/V1S:SV2” cells exposed to SiO_2_ and chitin nanoparticles plus 1 mM LLME. **(C)** The ratios of cells containing αS inclusions to total cells for each group in B) are statistically analyzed and shown as a bar graph. Error bars represent the standard error of the mean (**p < 0.01, compared with Con group; ^##^*p* < 0.01, comparing subsets linked by line, *n* = 3).

Since previous studies have shown that the effects of nanoparticles on αS aggregation are associated with their mutual binding affinity (Alvarez et al., 2013; Mohammadi and Nikkhah, 2017; Tahaei Gilan et al., 2019), we investigated if binding affinity plays a role in the formation of αS inclusions in cells after the internalization of nanoparticles. To answer this question, we designed an experiment that used nanoparticles with and without strong binding affinity to αS as positive and negative controls. Therefore, we compared the affinity to αS between nanoparticles from different materials and chose SiO_2_ and chitin nanoparticles with similar size (80 nm) as the two controls because binding studies showed that there was considerable αS bound to SiO_2_ nanoparticles, while only negligible αS bound to chitin nanoparticles (Figure 5A).

Next, we establish a new cell line that stably co-expresses chitin binding domain (CBD)-tagged V1S at N-terminus (CBD-V1S) and SV2 at C-terminus (SV2-CBD), referred to as “H4/CBD-V1S:SV2-CBD” (see Supplementary Figure 1). Due to the nature of CBD (Hashimoto et al., 2000), both CBD-V1S and SV2-CBD expressed in this cell line can specifically bind to exogenous chitin nanoparticles. If binding affinity to αS is a determining factor for the induction of αS inclusions by nanoparticles, H4/CBD-V1S:SV2-CBD cells with endolysosomal impairment should form αS inclusions upon the treatment with chitin nanoparticles and LLME because CBD fused αS is able to specifically bind chitin through its CBD tag. In contrast, the number of αS inclusions induced by chitin nanoparticles in H4/V1S:SV2 cell line with lysosomal impairment should be significantly less because there is only negligible binding between αS and chitin due to the absence of CBD. However, SiO_2_ nanoparticles should induce αS inclusions in both cell lines due to their significantly higher affinity to αS. Results from the cell-based study were exactly consistent with our expectations (see Figures 5B,C), strongly supporting the hypothesis that binding affinity of nanoparticles to αS determines their capability to induce αS inclusions in cells.

### Lysosomal Glucocerebrosidase Deficiency May Render Cells More Susceptible to αS Inclusions in Response to Nanoparticle Treatment

The effect of LLME on endolysosomal impairment prompted us to study if there were endogenous lysosomotropic substances in human cells that are associated with PD. We found sphingosine to be a potential candidate. Sphingosine is an endogenous biomolecule significantly increased in patients with Gaucher disease (GD) due to the Glucocerebrosidase (GCase) deficiency in this disease (Mistry et al., 2014). It is also a lysosomotropic reagent similar to LLME as its accumulation in cells leads to the formation of dilated endolysosomes (Lima et al., 2017). GD is the most common of the lysosomal storage diseases, and it is caused by a hereditary deficiency of the enzyme GCase, which is encoded by a gene named *GBA1*. Interestingly, mutation of *GBA1* gene recently emerged as a common genetic risk associated with PD. Approximately 5% of patients with PD carry a *GBA1* mutation, compared to <1% of the control population (Stoker et al., 2018). Moreover, a decrease in Gcase activity has been detected in idiopathic brain tissue of PD (Chiasserini et al., 2015; Parnetti et al., 2017). Therefore, we hypothesized that GCase deficiency may render cells more susceptible to the formation of αS inclusions in response to nanoparticle treatment.

**FIGURE 6:**
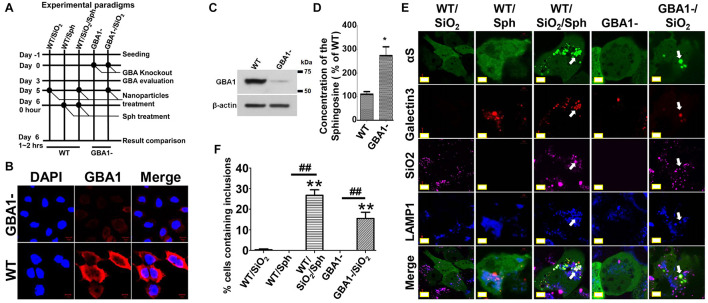
Glucocerebrosidase deficiency may render cells more susceptible to the formation of αS inclusions in response to nanoparticle treatment. **(A)** shows the experiment design. **(B–D)** Immunocytochemistry, immunoblotting, and sphingosine assays were employed to verify the loss of GCase and increase of sphingosine in GBA1- cells. The level of sphingosine (Sph) measured in cells with and without GBA1 deletion was statistically analyzed and shown as a bar graph. The difference between the GBA1- and WT is statistically significant. **(E)** After completion of different treatments as illustrated in (A), the five groups of cells, referred to as WT/SiO_2_, WT/Sph, WT/SiO_2_/Sph, GBA1- and GBA1-/SiO_2_, were fixed in 4% PFA and evaluated by a confocal microscopy. **(F)** The number of cells containing αS inclusions and the proportion of cells with inclusions were counted and tabulated for statistical analysis shown as a bar graph. Error bars represent the standard error of the mean (*p < 0.05, **p < 0.01, compared with WT/SiO_2_ group; ^##^*p* < 0.01, comparing subsets linked by line, *n* = 3).

To test this hypothesis, cells with *GBA1* gene deletion were generated and used to assess αS aggregation in response to nanoparticles. As shown in Figure 6A, H4/V1S:SV2/LAMP1-eCFP/mCherry-galectin-3 cells were infected with lentivirus carrying *GBA1*-knockout and control vectors, referred to as “GBA1-” and “WT.” After 3 days of infection, a portion of sibling cells from WT and GBA1- were harvested to evaluate the effect of GBA1 deletion on the level of GCase expression (Figures 6B,C) and sphingosine production (Figure 6D). A part of sibling cultures from “WT” and “GBA1-” groups were then treated with Alex Fluor^TM^ 647-labeled SiO_2_ nanoparticles to derive two subgroups, referred to as “WT/SiO_2_” and “GBA1-/SiO_2_.” After 1 day, “WT” cells with and without SiO_2_ nanoparticle treatment were exposed to sphingosine (Sph, 20 μM) to induce endolysosomal impairment, which further derived two more subgroups referred to as “WT/Sph” and “WT/SiO_2_/Sph.” An hour after sphingosine exposure, cells in all groups were fixed with 4% PFA and evaluated by confocal microscopy for the presence of αS inclusions. As expected, Sph treatment or *GBA1* deletion both induced endolysosomal membrane rupture, reflected by colocalization of LAMP-eCFP and punctate mCherry-galectin-3 signals in cells. No αS inclusions were observed in cells with either endolysosomal rupture induction (GBA1- or WT/Sph) or nanoparticle treatment alone (WT/SiO_2_). In contrast, cells with both nanoparticle treatment and endolysosomal impairment (WT/SiO_2_/Sph and GBA1-/SiO_2_) developed αS inclusions in numbers significantly different from the groups of WT/SiO_2_, GBA1-, and WT/Sph (Figures 6E,F). Since cells with *GBA1* deletion had a significantly higher level of sphingosine (Figure 6D), and either *GBA1* deletion or exogenous sphingosine treatment can induce endolysosomal membrane rupture and facilitate nanoparticles to induce the formation of αS inclusions, it is reasonable to conclude that GCase deficiency may render cells more susceptible to the formation of αS inclusions in response to nanoparticle treatment due to the high risk of impairment in endolysosomal system.

### Nanoparticle-Induced Inclusions Contain Membrane-Bound αS

**FIGURE 7:**
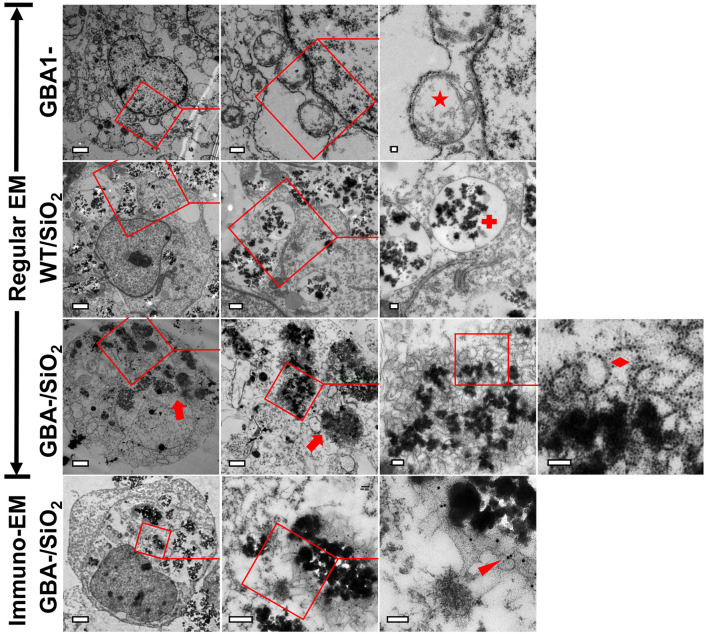
Nanoparticle-induced αS inclusions contained membrane-bound αS. Three groups of cells, WT/SiO_2_, GBA1-/SiO_2_, and GBA1-, described in Figure 6, were prepared for EM examination. Uranyl-lead EM staining revealed that only cells in GBA1-/SiO_2_ group contained abundant nanoparticle-associated inclusions (denoted by red arrows). The fields on the **left panels** were magnified in the middle panels and further in the **right panels** to reveal the presence of inclusions in the cells of GBA-/SiO_2_ group, and the inclusions contained a cluster of membranous structures in the absence of intact encircling membrane and filamentous structure (denoted by a red diamond), indicating that the endolysosomes in cells were ruptured and no αS fibril was formed. In contrast, cells in WT/SiO_2_ group contained abundant congregated nanoparticles which were well-confined in intact endolysosomes (denoted by a red cross), and those in GBA1- group contain abundant abnormally swollen membranous structures (denoted by a red star). Immuno-EM further showed that the nanoparticle-associated membranous structures in GBA1-/SiO_2_ group were immunolabeled by primary antibody against αS (NACP98, Mayo Clinic) (Dickson et al., 1999) and 18-nm gold conjugated secondary antibody (111-215-144, Jackson ImmunoResearch Laboratories) as denoted by a red arrowhead. Scale bar: 2 μm for the first column; 500 nm for the second column; 100 nm for the third and fourth column.

To understand the ultrastructure of nanoparticle-induced αS inclusions in cells with Gcase deficiency, cultures belonging to the groups WT/SiO_2_, GBA1-/SiO_2_, and GBA1- were processed for EM examination. Our observations (Figure 7) revealed that only cells in GBA1-/SiO_2_ group contained abundant nanoparticle-associated inclusions. In contrast, cells in WT/SiO_2_ group contained abundant aggregated nanoparticles within endolysosomes (denoted by red cross in Figure 7), consistent with the view that nanoparticles are internalized *via* endocytosis (Behzadi et al., 2017). Moreover, those in GBA1- group had abnormally swollen membranous structures (denoted by red star in Figure 7), similar to those observed in neurons from animals with *GBA1* knockout (Uemura et al., 2015; Schondorf et al., 2018). The inclusions detected in GBA1-/SiO_2_ group consisted of a mixture of congregated nanoparticles and fragmented membranous structures in the absence of intact encircling membrane and filamentous structure (denoted by red diamond in Figure 7), indicating that the endolysosomes in cells were ruptured and no αS fibril was formed.

Subsequent immuno-EM staining revealed that the nanoparticle-associated membranous structures were immunoreactive to antibody against αS (denoted by red arrowhead in Figure 7), suggesting that the membrane-bound αS is a constituent of the nanoparticle-induced inclusion.

### Exogenous αS Fibril-Induced αS Inclusions in Cells Contain Membrane-Bound αS

**FIGURE 8:**
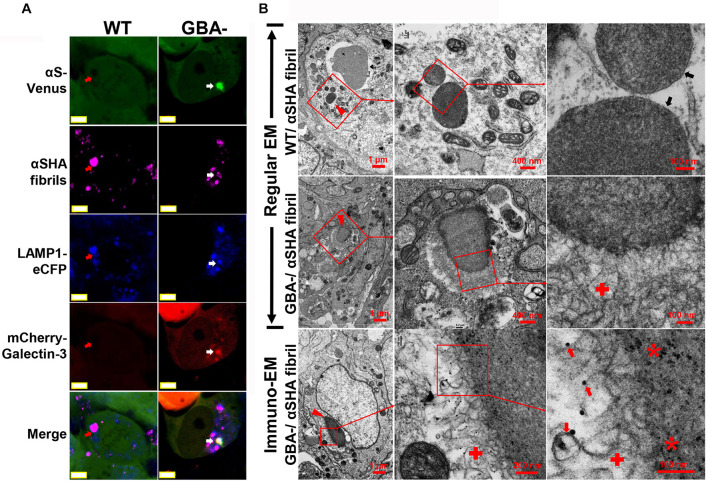
Exogenous αS fibril-induced αS inclusions in cells contain membrane-bound αS. H4/V1S:SV2/LAMP1-eCFP/mCherry-galectin-3 cells with GBA1 deletion (GBA1-) or without (WT) were treated with HA-tagged αS fibrils (αSHA) with reduced seeding ability by preincubation at 4°C for 2 days. **(A)** Representative confocal images showed that seeded inclusions were associated with membrane proteins, such as LAMP1 and galectin-3 reflected by their fused fluorescent protein eCFP and mCherry (denoted by white arrow), respectively. In contrast, seeds in WT cells were only colocalized with endolysosomal marker LAMP1 (denoted by red arrows). Scale bar: 5 μm. **(B)** Representative EM images showed internalized αSHA fibrils in cells (denoted by red arrowheads). In WT cells, they were only detained in endolysosomes (denoted by black arrows); in GBA- cells, they formed membrane-associated inclusions in which the seeds were enclosed by clustered membrane structures (denoted by red cross). Venus-immunopositive outer layer and HA-positive inner core from immuno-EM suggested that the membranous structures bear intracellular αS-Venus (denoted by red arrows) and the inner core is exogenous αSHA fibrils (denoted by red asterisk). The framed region in each picture was enlarged and shown on the right. Scale bars are shown in each picture.

As we know, αS fibrils can recruit unfolded αS to amplify aggregates in buffer system; moreover, exogenous αS fibrils can seed the formation of Lewy body-like intracellular inclusions in cultured cells (Luk et al., 2009). If membrane-bound αS is a constituent of nanoparticle-induced αS inclusions, it should also exist in αS fibril-induced inclusions due to the mutual affinity between αS fibrils and αS molecules. To find the answer, we used mature fibrils derived from recombinant αS fused with HA tag (αSHA) to treat H4/V1S:SV2/LAMP1-eCFP/mCherry-galectin-3 cells with GBA1 deletion (GBA1-) or without (WT). Cells of GBA1- should be more susceptible to the formation of αS inclusions due to the endolysosomal impairment compared with those of WT. Results from confocal imaging showed that seeded inclusions were associated with membrane proteins, such as LAMP1 and galectin-3 reflected by their fused fluorescent protein eCFP and mCherry (in Figure 8A), respectively, suggesting the involvement of membrane structures and membrane rupture in seeding. In contrast, seeds in WT cells were only colocalized with endolysosomal marker LAMP1, suggesting its endocytic pathway of cellular uptake, but did not induce αS inclusion due to the confinement of nanoparticles in endolysosomes. Results from EM showed that αSHA fibrils were internalized by cells. In WT cells, they were only detained in endolysosomes; In GBA- cells, they formed membrane-associated inclusions in which the seeds were enclosed by clustered membrane structures. Moreover, immuno-EM revealed that the inclusions consisted of Venus-immunopositive membranous outer layer and HA-immunopositive inner core (denoted by red asterisk), suggesting that these inclusions were formed by the seeding between exogenous αSHA fibrils and cytoplasmic membrane-bound αS-Venus.

## Discussion

Although over recent decades nanoparticle-based therapies have been explored as a potential tool for disease treatment, it still remains controversial if such strategy is applicable to the treatment of neurodegenerative diseases (Mushtaq et al., 2015; Naqvi et al., 2020). As we know, the accumulation of aggregated protein is a prominent feature of neurodegenerative diseases, and the aggregate-prone proteins, which are highly expressed in brain cells, could be sequestered by nanoparticles if there is mutual affinity. This brings up a question as to whether the accumulated proteins on the surface of nanoparticles could aggregate and become toxic. As for αS, the major causative protein in PD, ample evidence from studies in buffer system has shown that the promotive or inhibitive effects of nanoparticles on this protein are determined by multiple factors, such as shape, surface charge, and concentration (D’Onofrio et al., 2020; Pichla et al., 2020). However, there were only limited studies focusing on the effect of nanoparticles on αS aggregation in cellular and animal models.

In the present study, we demonstrated that nanoparticles can induce αS assembly to form inclusions upon internalization into cells with endolysosomal impairment. It is worth noting that in cell treatment, only a portion of nanoparticles can successfully enter into cells and then escape from endolysosome to interact with cytoplasmic αS; therefore, nanoparticles at low concentration might not result in the best effect. However, nanoparticles at too high concentration should also be avoided because cells overwhelmed by nanoparticles may become unhealthy. This is very different from experiments in a buffer system in which the concentration of nanoparticles for the induction of αS aggregation can be used in a wide range (Vitali et al., 2018; Pang et al., 2021). Therefore, the concentration of nanoparticles should be carefully optimized for cell treatment.

Although a previous study by Xie and Wu (2016) showed that SiO_2_ nanoparticles can induce αS aggregates in PC12 cells, our study is the first report showing that nanoparticles can escape from ruptured endolysosomes (reflected by LAMP1-associated punctate galectin-3) to directly interact with cytoplasmic αS, leading to the formation of αS inclusions in cells with the impaired endolysosomal system. In the study by Xie and Wu (2016) nanoparticles were confined in endolysosomes and consequently not associated with detergent-insoluble αS, but there was an increase in αS levels in cells treated with nanoparticles compared with those without. They hypothesized that this was due to nanoparticle-elicited oxidative stress and inhibition of the ubiquitin-proteasomal system. To our knowledge, the present study is the first to show that nanoparticles may directly interact with cytoplasmic αS to form toxic aggregates.

Even though nanoparticle-induced αS inclusions in our cell models contained detergent-resistant and pathologic forms of αS aggregates, no filamentous αS structures were observed with immunoEM (Figures 4, 7). Therefore, the αS inclusions in our cell models should be formed at an early stage of αS aggregation. The formation of filamentous structures may require a longer duration of treatment, higher levels of αS expression, or other unknown cellular factors. It is unclear how αS aggregates upon contact with nanoparticles. A mechanism previously proposed for the aggregation of membrane-bound αS may provide a reasonable explanation (Galvagnion et al., 2015). In that model, αS molecules normally bind and accumulate on membrane surfaces leading to locally high concentrations. Localized high concentration of αS may promote conformational changes that favor nucleation, which triggers a cascade of events leading to high molecular weight aggregates. If nanoparticles can bind αS, it might be predicted that they may sequester αS on the nanoparticle surface, which can favor nucleation and subsequent aggregation once a critical concentration is reached. This hypothesis was supported by experiments in which chitin nanoparticles induced the formation of αS inclusions in cells of “H4/CBD-V1S:SV2-CBD” due to sequestration of CBD-fused αS *via* the specific binding affinity between chitin and CBD tag. In contrast, “H4/V1S:SV2” cells lacking the CBD, the binding partner of chitin, did not lead to aggregation (Figure 5). Therefore, it is reasonable to suggest that exogenous or endogenous substances with binding affinity for αS, once in contact with cytoplasmic αS, may promote αS pathology in humans.

We found that endolysosomal impairment is critical in the susceptibility of cells to nanoparticle-induced formation of αS inclusions. This is supported by the fact that cultured cells can develop nanoparticle-induced αS inclusions only if their endolysosomal system is disrupted by treatment with lysosomotropic agents or GCase deficiency (Figures 2, 6). In this regard, we further demonstrated that sphingosine, an endogenous lysosomotropic biomolecule, plays an important role in determining the susceptibility of cells to αS inclusions in the presence of nanoparticles. In these cells, increased sphingosine levels due to *GBA1* deletion or GCase deficiency impair endolysosomal integrity (Figures 6, 7). It is worth noting that GCase deficiency is associated with the accumulation of multiple lipid metabolites (Abed Rabbo et al., 2021), and sphingosine may not be the only critical factor in this process.

In addition, we found that smaller-sized nanoparticles were more effective in inducing the formation of αS inclusions (Figure 3). Size-dependent phenomenon may be related to the possibility that smaller nanoparticles more readily escape from endolysosomes. Alternatively, nanoparticles of smaller size have higher curvature on surface, which is more likely to promote protein aggregation, as shown in previous studies on the impact of membrane curvature on amyloid aggregation (Terakawa et al., 2018). Given these observations, caution needs to be taken when using nanoparticles in the treatment of PD, since lysosomal impairment has been considered to play an important role in PD and related diseases (Wang et al., 2018). Moreover, for the selection of nanoparticles, the size, the nature of material, and the binding affinity to αS on the surface should also be considered.

**FIGURE 9:**
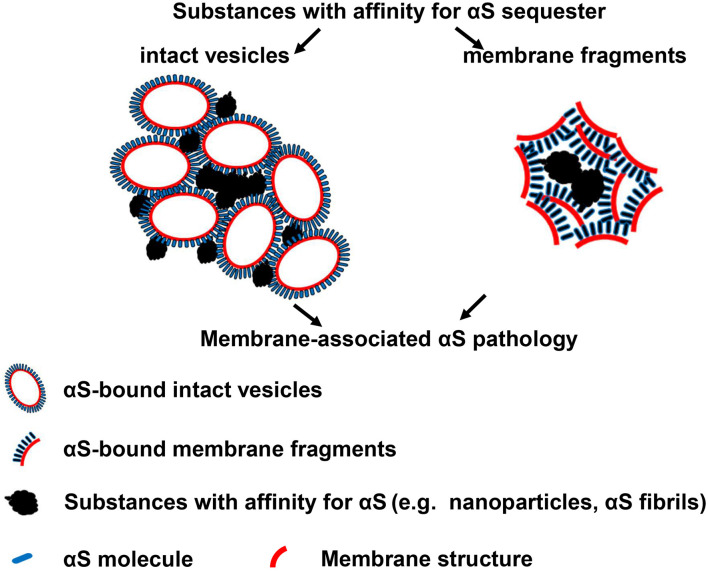
A Hypothesis: substances with affinity for αS sequester membrane-bound αS to form membrane-associated αS pathology. **Left:** sequestration of membrane-bound αS with intact vesicles; **Right:** sequestration of membrane-bound αS with fragmented membranes.

It is interesting that nanoparticle-induced αS inclusions contain a cluster of membranous structures at the ultrastructural level. This result is consistent with the finding reported by Shahmoradian et al. (2019) that Lewy pathology in PD consists of crowded lipid membranes. More importantly, such membranous structures can be immunolabeled by antibody against αS, indicative of membrane-bound αS (Figure 7). This result suggests a role of membranous structures in the formation of αS pathology because membrane-bound αS has a higher propensity for aggregation into higher-order oligomers/aggregates (Lee et al., 2002; Burre et al., 2014). Although Shahmoradian et al. hypothesized that “lipid membrane fragments and distorted organelles together with a non-fibrillar form of αS are the main structural building blocks for the formation of Lewy pathology” (Shahmoradian et al., 2019), our results raised another possibility that a certain type of membranous structures, due to bearing high concentration of bound αS, may be recruited as byproducts by αS-affinitive substance (e.g., SiO_2_ nanoparticles in the present study) to become an important constituent of αS pathology. If this is the case, the crowded lipid membranous structures observed in αS pathology in the brain, which has been considered mainly due to impaired organellar trafficking in previous studies (Hunn et al., 2015; Shahmoradian et al., 2019), could also result from sequestration of αS by substances with strong binding affinity to αS. Since unfolded αS can be recruited and templated by αS fibrils leading to the propagation of αS aggregates (Wood et al., 1999), it is reasonable to consider αS fibrils as a type of substances with strong binding affinitive to αS. Accordingly, αS inclusions induced by αS fibrils should also contain αS-associated membranes. Indeed, this speculation was confirmed by our cell-based study. As shown in Figure 8, cells with *GBA1* deletion and αS fibrils treatment can form αS inclusions associated with membrane proteins, such as LAMP1 and galectin-3, and these inclusions also contained clustered membranous structures and membrane-bound αS at the ultrastructural level. Based on these results, we proposed that sequestration of membrane-bound αS by substances with binding affinity for αS (e.g., nanoparticles, αS filaments) could contribute to the formation of membrane-associated αS pathology. A schematic picture of this hypothesis is shown in Figure 9.

Overall, this study investigated the effect of nanoparticles with affinity for αS on αS aggregation in different cell models and made the novel observation that loss of endolysosomal integrity and the intrinsic binding affinity of the nanoparticles induced the sequestration of cytoplasmic αS. Furthermore, we propose a new mechanism to explain the role of crowded lipid membranous structures in Lewy pathology. This study not only provides support for the potential risk of nanoparticles in the treatment of neurologic disorders, especially for neurodegenerative diseases, such as PD and multiple system atrophy, which are associated with aggregation-prone αS, but it also advances understanding about mechanisms underlying the formation of αS pathology in PD.

## Data Availability Statement

The original contributions presented in the study are included in the article/Supplementary Material, further inquiries can be directed to the corresponding authors.

## Author Contributions

PJ created the ideas, designed and conducted the experiments, and wrote the manuscript. MG performed the data analysis and image labeling. S-HY and DD revised the manuscript. All authors contributed to the article and approved the submitted version.

## Conflict of Interest

The authors declare that the research was conducted in the absence of any commercial or financial relationships that could be construed as a potential conflict of interest.

## Publisher’s Note

All claims expressed in this article are solely those of the authors and do not necessarily represent those of their affiliated organizations, or those of the publisher, the editors and the reviewers. Any product that may be evaluated in this article, or claim that may be made by its manufacturer, is not guaranteed or endorsed by the publisher.

## References

[B1] Abed Rabbo M. Khodour Y. Kaguni L. S. Stiban J. (2021). Sphingolipid lysosomal storage diseases: from bench to bedside. *Lipids Health Dis.* 20:44. 10.1186/s12944-021-01466-0 33941173 PMC8094529

[B2] Alvarez Y. D. Fauerbach J. A. Pellegrotti J. V. Jovin T. M. Jares-Erijman E. A. Stefani F. D. (2013). Influence of gold nanoparticles on the kinetics of alpha-synuclein aggregation. *Nano Lett.* 13 6156–6163. 10.1021/nl403490e 24219503

[B3] Bae E. J. Yang N. Y. Lee C. Lee H. J. Kim S. Sardi S. P. (2015). Loss of glucocerebrosidase 1 activity causes lysosomal dysfunction and alpha-synuclein aggregation. *Exp. Mol. Med.* 47:e153. 10.1038/emm.2014.128 25813221 PMC4351412

[B4] Behzadi S. Serpooshan V. Tao W. Hamaly M. A. Alkawareek M. Y. Dreaden E. C. (2017). Cellular uptake of nanoparticles: journey inside the cell. *Chem. Soc. Rev.* 46 4218–4244. 10.1039/C6CS00636A 28585944 PMC5593313

[B5] Bernal-Conde L. D. Ramos-Acevedo R. Reyes-Hernandez M. A. Balbuena-Olvera A. J. Morales-Moreno I. D. Arguero-Sanchez R. (2019). Alpha-synuclein physiology and pathology: a perspective on cellular structures and organelles. *Front. Neurosci.* 13:1399. 10.3389/fnins.2019.01399 32038126 PMC6989544

[B6] Burre J. Sharma M. Sudhof T. C. (2014). alpha-Synuclein assembles into higher-order multimers upon membrane binding to promote SNARE complex formation. *Proc. Natl. Acad. Sci. U.S.A.* 111 E4274–E4283. 10.1073/pnas.1416598111 25246573 PMC4210039

[B7] Chiasserini D. Paciotti S. Eusebi P. Persichetti E. Tasegian A. Kurzawa-Akanbi M. (2015). Selective loss of glucocerebrosidase activity in sporadic Parkinson’s disease and dementia with Lewy bodies. *Mol. Neurodegener.* 10:15. 10.1186/s13024-015-0010-2 25881142 PMC4428238

[B8] Dehay B. Bove J. Rodriguez-Muela N. Perier C. Recasens A. Boya P. (2010). Pathogenic lysosomal depletion in Parkinson’s disease. *J. Neurosci.* 30 12535–12544. 10.1523/JNEUROSCI.1920-10.2010 20844148 PMC6633458

[B9] Dehay B. Martinez-Vicente M. Ramirez A. Perier C. Klein C. Vila M. (2012). Lysosomal dysfunction in Parkinson disease: ATP13A2 gets into the groove. *Autophagy* 8 1389–1391. 10.4161/auto.21011 22885599 PMC3442887

[B10] Dickson D. W. Liu W. Hardy J. Farrer M. Mehta N. Uitti R. (1999). Widespread alterations of alpha-synuclein in multiple system atrophy. *Am. J. Pathol.* 155 1241–1251. 10.1016/S0002-9440(10)65226-1 10514406 PMC1867032

[B11] D’Onofrio M. Munari F. Assfalg M. (2020). Alpha-synuclein-nanoparticle interactions: understanding, controlling and exploiting conformational plasticity. *Molecules* 25:5625. 10.3390/molecules25235625 33260436 PMC7731430

[B12] Flavin W. P. Bousset L. Green Z. C. Chu Y. Skarpathiotis S. Chaney M. J. (2017). Endocytic vesicle rupture is a conserved mechanism of cellular invasion by amyloid proteins. *Acta Neuropathol.* 134 629–653. 10.1007/s00401-017-1722-x 28527044

[B13] Galvagnion C. Buell A. K. Meisl G. Michaels T. C. Vendruscolo M. Knowles T. P. (2015). Lipid vesicles trigger alpha-synuclein aggregation by stimulating primary nucleation. *Nat. Chem. Biol.* 11 229–234. 10.1038/nchembio.1750 25643172 PMC5019199

[B14] Gavhane Y. N. Yadav A. V. (2012). Loss of orally administered drugs in GI tract. *Saudi Pharm. J.* 20 331–344. 10.1016/j.jsps.2012.03.005 23960808 PMC3744959

[B15] Hashimoto M. Ikegami T. Seino S. Ohuchi N. Fukada H. Sugiyama J. (2000). Expression and characterization of the chitin-binding domain of chitinase A1 from *Bacillus circulans* WL-12. *J. Bacteriol.* 182 3045–3054. 10.1128/JB.182.11.3045-3054.2000 10809681 PMC94488

[B16] Hata K. Higashisaka K. Nagano K. Mukai Y. Kamada H. Tsunoda S. (2014). Evaluation of silica nanoparticle binding to major human blood proteins. *Nanoscale Res. Lett.* 9:2493. 10.1186/1556-276X-9-668 26089000 PMC4493834

[B17] Hersh D. S. Wadajkar A. S. Roberts N. Perez J. G. Connolly N. P. Frenkel V. (2016). Evolving drug delivery strategies to overcome the blood brain barrier. *Curr. Pharm. Des.* 22 1177–1193. 10.2174/1381612822666151221150733 26685681 PMC4900538

[B18] Hoshyar N. Gray S. Han H. Bao G. (2016). The effect of nanoparticle size on in vivo pharmacokinetics and cellular interaction. *Nanomedicine* 11 673–692. 10.2217/nnm.16.5 27003448 PMC5561790

[B19] Hunn B. H. Cragg S. J. Bolam J. P. Spillantini M. G. Wade-Martins R. (2015). Impaired intracellular trafficking defines early Parkinson’s disease. *Trends Neurosci.* 38 178–188. 10.1016/j.tins.2014.12.009 25639775 PMC4740565

[B20] Jiang P. Gan M. Ebrahim A. S. Castanedes-Casey M. Dickson D. W. Yen S. H. (2013). Adenosine monophosphate-activated protein kinase overactivation leads to accumulation of alpha-synuclein oligomers and decrease of neurites. *Neurobiol. Aging* 34 1504–1515. 10.1016/j.neurobiolaging.2012.11.001 23200460 PMC3570625

[B21] Jiang P. Gan M. Yen S. H. McLean P. J. Dickson D. W. (2017). Impaired endo-lysosomal membrane integrity accelerates the seeding progression of alpha-synuclein aggregates. *Sci. Rep.* 7:7690. 10.1038/s41598-017-08149-w 28794446 PMC5550496

[B22] Jiang P. Ko L. W. Jansen K. R. Golde T. E. Yen S. H. (2008). Using leucine zipper to facilitate alpha-synuclein assembly. *FASEB J.* 22 3165–3174. 10.1096/fj.08-108365 18492724 PMC2518257

[B23] Joshi N. Basak S. Kundu S. De G. Mukhopadhyay A. Chattopadhyay K. (2015). Attenuation of the early events of alpha-synuclein aggregation: a fluorescence correlation spectroscopy and laser scanning microscopy study in the presence of surface-coated Fe3O4 nanoparticles. *Langmuir* 31 1469–1478. 10.1021/la503749e 25561279

[B24] Khodabandeh A. Y. Yakhchian R. Hasan A. Paray B. A. Shahi F. Rasti B. (2020). Silybin as a potent inhibitor of a-synuclein aggregation and associated cytotoxicity against neuroblastoma cells induced by zinc oxide nanoparticles. *J. Mol. Liquids* 310:113198. 10.1016/j.molliq.2020.113198

[B25] Ko L. W. Ko H. H. Lin W. L. Kulathingal J. G. Yen S. H. (2008). Aggregates assembled from overexpression of wild-type alpha-synuclein are not toxic to human neuronal cells. *J. Neuropathol. Exp. Neurol.* 67 1084–1096. 10.1097/NEN.0b013e31818c3618 18957893 PMC2768257

[B26] Lafuente J. V. Requejo C. Ugedo L. (2019). Nanodelivery of therapeutic agents in Parkinson’s disease. *Prog. Brain Res.* 245 263–279. 10.1016/bs.pbr.2019.03.004 30961870

[B27] Lee H. J. Choi C. Lee S. J. (2002). Membrane-bound alpha-synuclein has a high aggregation propensity and the ability to seed the aggregation of the cytosolic form. *J. Biol. Chem.* 277 671–678. 10.1074/jbc.M107045200 11679584

[B28] Leyva-Gomez G. Cortes H. Magana J. J. Leyva-Garcia N. Quintanar-Guerrero D. Floran B. (2015). Nanoparticle technology for treatment of Parkinson’s disease: the role of surface phenomena in reaching the brain. *Drug Discov. Today* 20 824–837. 10.1016/j.drudis.2015.02.009 25701281

[B29] Lima S. Milstien S. Spiegel S. (2017). Sphingosine and Sphingosine Kinase 1 Involvement in Endocytic Membrane Trafficking. *J. Biol. Chem.* 292 3074–3088. 10.1074/jbc.M116.762377 28049734 PMC5336145

[B30] Linse S. Cabaleiro-Lago C. Xue W. F. Lynch I. Lindman S. Thulin E. (2007). Nucleation of protein fibrillation by nanoparticles. *Proc. Natl. Acad. Sci. U.S.A.* 104 8691–8696. 10.1073/pnas.0701250104 17485668 PMC1866183

[B31] Luk K. C. Song C. O’Brien P. Stieber A. Branch J. R. Brunden K. R. (2009). Exogenous alpha-synuclein fibrils seed the formation of Lewy body-like intracellular inclusions in cultured cells. *Proc. Natl. Acad. Sci. U.S.A.* 106 20051–20056. 10.1073/pnas.0908005106 19892735 PMC2785290

[B32] Mahapatro A. Singh D. K. (2011). Biodegradable nanoparticles are excellent vehicle for site directed in-vivo delivery of drugs and vaccines. *J. Nanobiotechnol.* 9:55. 10.1186/1477-3155-9-55 22123084 PMC3238292

[B33] Meissner W. G. Frasier M. Gasser T. Goetz C. G. Lozano A. Piccini P. (2011). Priorities in Parkinson’s disease research. *Nat. Rev. Drug Discov.* 10 377–393. 10.1038/nrd3430 21532567

[B34] Mhyre T. R. Boyd J. T. Hamill R. W. Maguire-Zeiss K. A. (2012). Parkinson’s disease. *Subcell. Biochem.* 65 389–455. 10.1007/978-94-007-5416-4_16 23225012 PMC4372387

[B35] Mistry P. K. Liu J. Sun L. Chuang W. L. Yuen T. Yang R. (2014). Glucocerebrosidase 2 gene deletion rescues type 1 Gaucher disease. *Proc. Natl. Acad. Sci. U.S.A.* 111 4934–4939. 10.1073/pnas.1400768111 24639522 PMC3977292

[B36] Mohammadi S. Nikkhah M. (2017). TiO2 nanoparticles as potential promoting agents of fibrillation of alpha-Synuclein, a Parkinson’s Disease-Related Protein. *Iran. J. Biotechnol.* 15 87–94. 10.15171/ijb.1519 29845055 PMC5811058

[B37] Moors T. Paciotti S. Chiasserini D. Calabresi P. Parnetti L. Beccari T. (2016). Lysosomal dysfunction and alpha-synuclein aggregation in Parkinson’s disease: diagnostic links. *Mov. Disord.* 31 791–801. 10.1002/mds.26562 26923732

[B38] Murthy S. K. (2007). Nanoparticles in modern medicine: state of the art and future challenges. *Int. J. Nanomed.* 2 129–141. PMC2673971 17722542

[B39] Murugadoss S. Lison D. Godderis L. Van Den Brule S. Mast J. Brassinne F. (2017). Toxicology of silica nanoparticles: an update. *Arch. Toxicol.* 91 2967–3010. 10.1007/s00204-017-1993-y 28573455 PMC5562771

[B40] Mushtaq G. Khan J. A. Joseph E. Kamal M. A. (2015). Nanoparticles, neurotoxicity and neurodegenerative diseases. *Curr. Drug Metab.* 16 676–684. 10.2174/1389200216666150812122302 26264205

[B41] Napierska D. Thomassen L. C. Lison D. Martens J. A. Hoet P. H. (2010). The nanosilica hazard: another variable entity. *Part. Fibre Toxicol.* 7:39. 10.1186/1743-8977-7-39 21126379 PMC3014868

[B42] Naqvi S. Panghal A. Flora S. J. S. (2020). Nanotechnology: a promising approach for delivery of neuroprotective drugs. *Front. Neurosci.* 14:494. 10.3389/fnins.2020.00494 32581676 PMC7297271

[B43] Pang C. Zhang N. Falahati M. (2021). Acceleration of alpha-synuclein fibril formation and associated cytotoxicity stimulated by silica nanoparticles as a model of neurodegenerative diseases. *Int. J. Biol. Macromol.* 169 532–540. 10.1016/j.ijbiomac.2020.12.130 33352154

[B44] Parnetti L. Paciotti S. Eusebi P. Dardis A. Zampieri S. Chiasserini D. (2017). Cerebrospinal fluid beta-glucocerebrosidase activity is reduced in Parkinson’s disease patients. *Mov. Disord.* 32 1423–1431. 10.1002/mds.27136 28843015

[B45] Pichla M. Bartosz G. Sadowska-Bartosz I. (2020). The antiaggregative and antiamyloidogenic properties of nanoparticles: a promising tool for the treatment and diagnostics of neurodegenerative diseases. *Oxid. Med. Cell. Longev.* 2020:3534570. 10.1155/2020/3534570 33123310 PMC7582079

[B46] Raza C. Anjum R. Shakeel N. U. A. (2019). Parkinson’s disease: mechanisms, translational models and management strategies. *Life Sci.* 226 77–90. 10.1016/j.lfs.2019.03.057 30980848

[B47] Schondorf D. C. Ivanyuk D. Baden P. Sanchez-Martinez A. De Cicco S. Yu C. (2018). The NAD+ precursor nicotinamide riboside rescues mitochondrial defects and neuronal Loss in iPSC and fly models of Parkinson’s Disease. *Cell Rep.* 23 2976–2988. 10.1016/j.celrep.2018.05.009 29874584

[B48] Shahmoradian S. H. Lewis A. J. Genoud C. Hench J. Moors T. E. Navarro P. P. (2019). Lewy pathology in Parkinson’s disease consists of crowded organelles and lipid membranes. *Nat. Neurosci.* 22 1099–1109. 10.1038/s41593-019-0423-2 31235907

[B49] Shang L. Nienhaus K. Nienhaus G. U. (2014). Engineered nanoparticles interacting with cells: size matters. *J. Nanobiotechnol.* 12:5. 10.1186/1477-3155-12-5 24491160 PMC3922601

[B50] Shen S. Wu Y. Liu Y. Wu D. (2017). High drug-loading nanomedicines: progress, current status, and prospects. *Int. J. Nanomed.* 12 4085–4109. 10.2147/IJN.S132780 28615938 PMC5459982

[B51] Stoker T. B. Torsney K. M. Barker R. A. (2018). “Pathological mechanisms and clinical aspects of GBA1 mutation-associated Parkinson’s disease,” in *Parkinson’s Disease: Pathogenesis and Clinical Aspects*, eds Stoker T. B. Greenland J. C. (Brisbane: Codon Publications). 30702840

[B52] Tahaei Gilan S. S. Yahya Rayat D. Mustafa T. A. Aziz F. M. Shahpasand K. Akhtari K. (2019). alpha-synuclein interaction with zero-valent iron nanoparticles accelerates structural rearrangement into amyloid-susceptible structure with increased cytotoxic tendency. *Int. J. Nanomed.* 14 4637–4648. 10.2147/IJN.S212387 31417259 PMC6602305

[B53] Teleanu D. M. Chircov C. Grumezescu A. M. Volceanov A. Teleanu R. I. (2018). Blood-brain delivery methods using nanotechnology. *Pharmaceutics* 10:269. 10.3390/pharmaceutics10040269 30544966 PMC6321434

[B54] Terakawa M. S. Lin Y. Kinoshita M. Kanemura S. Itoh D. Sugiki T. (2018). Impact of membrane curvature on amyloid aggregation. *Biochim. Biophys. Acta Biomembr.* 1860 1741–1764. 10.1016/j.bbamem.2018.04.012 29709613 PMC6205921

[B55] Uchimoto T. Nohara H. Kamehara R. Iwamura M. Watanabe N. Kobayashi Y. (1999). Mechanism of apoptosis induced by a lysosomotropic agent, L-Leucyl-L-Leucine methyl ester. *Apoptosis* 4 357–362. 10.1023/A:1009695221038 14634338

[B56] Uemura N. Koike M. Ansai S. Kinoshita M. Ishikawa-Fujiwara T. Matsui H. (2015). Viable neuronopathic Gaucher disease model in Medaka (*Oryzias latipes*) displays axonal accumulation of alpha-synuclein. *PLoS Genet.* 11:e1005065. 10.1371/journal.pgen.1005065 25835295 PMC4383526

[B57] Vitali M. Rigamonti V. Natalello A. Colzani B. Avvakumova S. Brocca S. (2018). Conformational properties of intrinsically disordered proteins bound to the surface of silica nanoparticles. *Biochim. Biophys. Acta Gen. Subj.* 1862 1556–1564. 10.1016/j.bbagen.2018.03.026 29621630

[B58] Wang C. Telpoukhovskaia M. A. Bahr B. A. Chen X. Gan L. (2018). Endo-lysosomal dysfunction: a converging mechanism in neurodegenerative diseases. *Curr. Opin. Neurobiol.* 48 52–58. 10.1016/j.conb.2017.09.005 29028540

[B59] Wood S. J. Wypych J. Steavenson S. Louis J. C. Citron M. Biere A. L. (1999). alpha-synuclein fibrillogenesis is nucleation-dependent. Implications for the pathogenesis of Parkinson’s disease. *J. Biol. Chem.* 274 19509–19512. 10.1074/jbc.274.28.19509 10391881

[B60] Xie H. Wu J. (2016). Silica nanoparticles induce alpha-synuclein induction and aggregation in PC12-cells. *Chem. Biol. Interact.* 258 197–204. 10.1016/j.cbi.2016.09.006 27613482

