# Supplementary material for: Nanoparticles With Affinity for α-Synuclein Sequester α-Synuclein to Form Toxic Aggregates in Neurons With Endolysosomal Impairment
Source: Front Mol Neurosci. 2021 Oct 20;14:738535. doi: 10.3389/fnmol.2021.738535 (PMC8565355; doi:10.3389/fnmol.2021.738535)
Supplement: Supplementary file 1 [file Data_Sheet_1.pdf]

Nanoparticles with affinity for α-synuclein sequester α-synuclein to form toxic aggregates in neurons with endolysosomal impairment

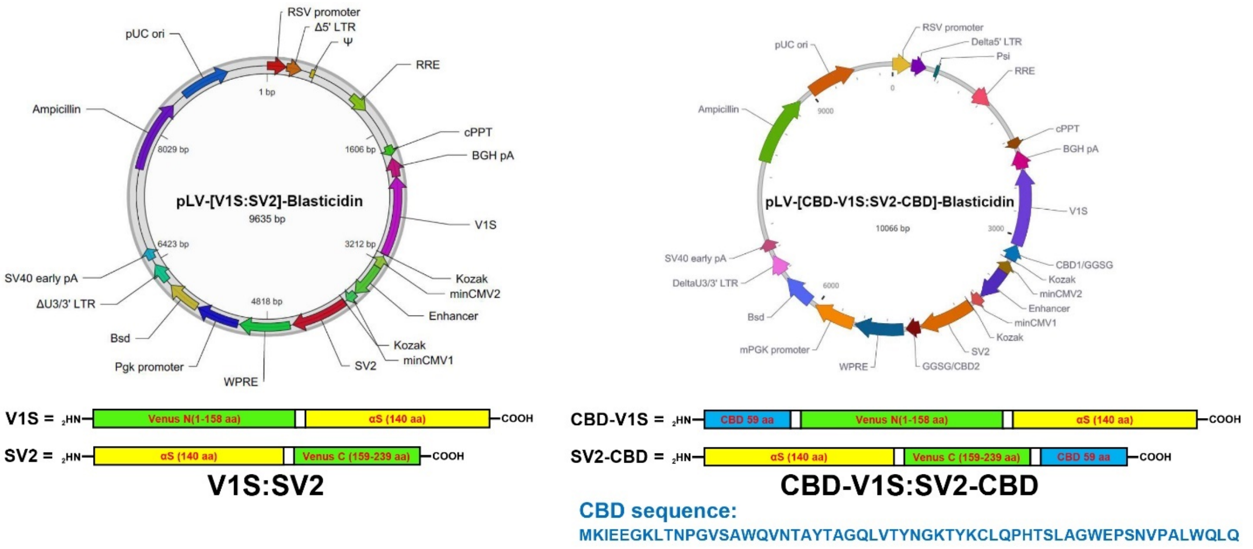

Figure S1. The maps of vectors encoding proteins of V1S:SV2 and CBD-V1S:SV2-CBD and the schematic of each protein.

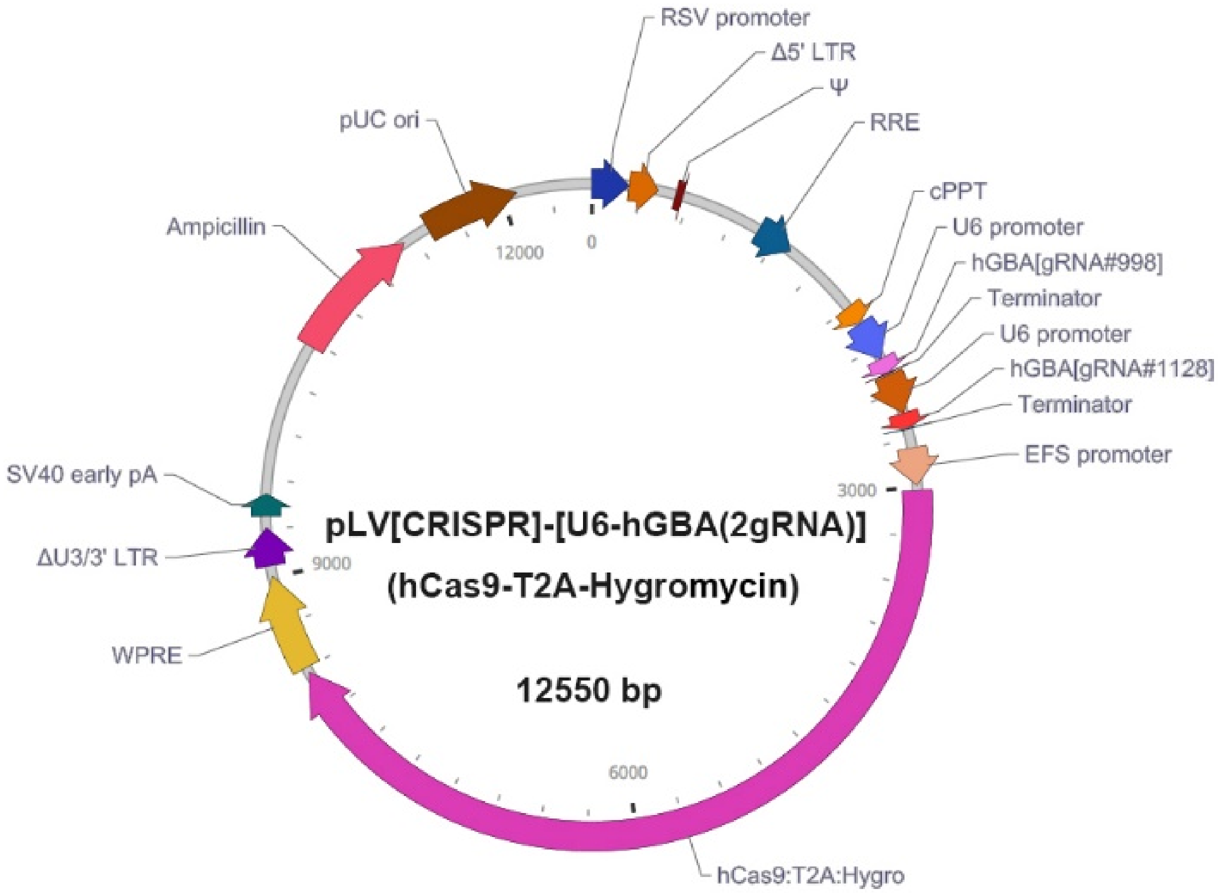

Figure S2. The map of vector for knockout of human GBA1 gene via CRISPR/Cas9 technology. The sequences of two single guide RNAs (sgRNAs) 998 and 1128 were “AGACCAATGGAGCGGTGAAT” and “TGTGGTGAGTACTGTTGGCG”, respectively.
